# Weakly encoded memories due to acute sleep restriction can be rescued after one night of recovery sleep

**DOI:** 10.1038/s41598-020-58496-4

**Published:** 2020-01-29

**Authors:** Daniel Baena, Jose L. Cantero, Lluís Fuentemilla, Mercedes Atienza

**Affiliations:** 1 Laboratory of Functional Neuroscience, Pablo de Olavide University, Seville, 41013 Spain; 2 CIBERNED, Network Center for Biomedical Research in Neurodegenerative Diseases, Madrid, Spain; 3 Cognition and Brain Plasticity Group, Bellvitge Biomedical Research Institute (IDIBELL), Hospitalet de Llobregat, 08907 Spain; 4 Department of Cognition, Development and Educational Psychology, University of Barcelona, Barcelona, 08035 Spain

**Keywords:** Consolidation, Human behaviour

## Abstract

Sleep is thought to play a complementary role in human memory processing: sleep loss impairs the formation of new memories during the following awake period and, conversely, normal sleep promotes the strengthening of the already encoded memories. However, whether sleep can strengthen deteriorated memories caused by insufficient sleep remains unknown. Here, we showed that sleep restriction in a group of participants caused a reduction in the stability of EEG activity patterns across multiple encoding of the same event during awake, compared with a group of participants that got a full night’s sleep. The decrease of neural stability patterns in the sleep-restricted group was associated with higher slow oscillation-spindle coupling during a subsequent night of normal sleep duration, thereby suggesting the instantiation of restorative neural mechanisms adaptively supporting cognition and memory. Importantly, upon awaking, the two groups of participants showed equivalent retrieval accuracy supported by subtle differences in the reinstatement of encoding-related activity: it was longer lasting in sleep-restricted individuals than in controls. In addition, sustained reinstatement over time was associated with increased coupling between spindles and slow oscillations. Taken together, these results suggest that the strength of prior encoding might be an important moderator of memory consolidation during sleep. Supporting this view, spindles nesting in the slow oscillation increased the probability of correct recognition only for weakly encoded memories. Current results demonstrate the benefit that a full night’s sleep can induce to impaired memory traces caused by an inadequate amount of sleep.

## Introduction

Healthy sleep is essential for optimal cognitive functioning. It seems to play a complementary role in human memory. While some of the studies indicate that sleep disruption reduces hippocampal activation during encoding in the awake period, leading to impaired memory retrieval after one night of recovery sleep^1,2^; other studies show that sleep mostly facilitates the consolidation of weaker memories^3–12^, thereby suggesting that memory consolidation during sleep is adaptive and prioritizes memories most vulnerable to forgetting. However, whether normal sleep could have a restorative impact on memories that are weakly encoded due to insufficient sleep in the previous night remains unknown.

**Figure 1:**
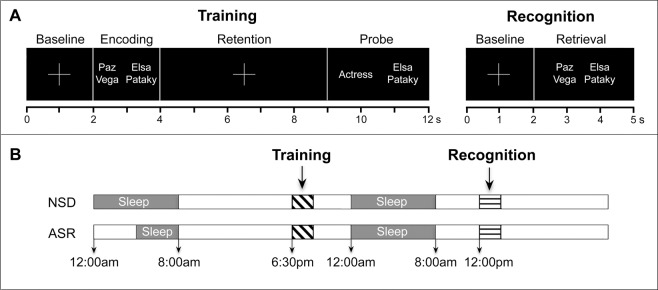
An overview of the memory task and experimental protocol. (**A**) Schematic illustration of the trial structure during the training and recognition phase. In the training task, participants are presented with two famous faces for 2 s. In the trial example, faces of Paz Vega and Elsa Pataky were shown together. After a retention period of 5 s, participants are asked to indicate if the profession and face correspond with the faces they have previously encountered. In the recognition task, participants must indicate whether or not they have seen this particular combination of faces. (**B**) Participants were trained in the evening (6:30 pm) following a night of either normal sleep duration (NSD; from 12:00 am to 8:00 am) or acute sleep restriction (ASR; from 4:00 am to 8:00 am). Memory recognition was tested at noon (12:00 pm) after a regular night of sleep (from 12:00 am to 8:00 am). EEG was recorded during sleep in the two consecutive nights as well as during the training and recognition task.

To address this question, we trained two groups of young healthy participants to associate faces of celebrities (Fig. 1A) after allowing a group of them to sleep normally for 8 h (normal sleep duration group; NSD), and after limiting sleep to 4 h by applying a bedtime delay procedure in another group (acute sleep restriction group; ASR) (Fig. 1B). To account for whether a recovery full night’s sleep influenced memory consolidation as a function of their strength, participants performed a recognition task one day after training. The simultaneous acquisition of EEG activity during all experimental sessions (including training, sleep, and retrieval) and the implementation of a time-resolved neural similarity analysis at training and retrieval^13,14^ allowed us to test several predictions.

Firstly, we addressed whether sleep restriction has an impact on strength of memories encoded in the subsequent awake period. Neural stability has been proposed to provide an index of memory strength^15–17^ that can be quantified by the degree to which neural patterns elicited by a given stimulus persist over repeated presentations^16^. Using functional magnetic resonance imaging (fMRI), previous research has shown that neural patterns in the higher visual cortex^18^ and regions that feed into the hippocampus^19^ are less stable across repetitions following total sleep deprivation. Accordingly, we hypothesized that sleep restriction would also impact the ability of the ASR group to elicit strong neural representations over repeated presentations of the same encoded pair of faces, and that this would be reflected as a decrease in the item-related neural representational stability when compared to the NSD group.

Secondly, we examined whether the interplay of neural oscillations supporting memory consolidation during sleep is associated with participants’ ability to elicit stable EEG activity patterns across encoding repetitions and with their capacity of correctly recognizing learned associations the next day. To this aim, we analyzed the temporal grouping of fast spindles (SPs; 13–16 Hz) by the depolarized up-state of slow oscillations (SOs; 0.5–4 Hz) during slow-wave sleep (SWS), which has been proposed as a key mechanism of overnight memory consolidation^20–24^. In particular, we tested whether the degree of SO-SP coupling during SWS is associated with the strength of memory encoding during the previous awake period, and to what extent this association predicts performance in the recognition task. Based on the assumption that sleep preferentially consolidates memories that have been poorly encoded during the previous waking period^3,4,7,8^, we hypothesized that the capacity of SO-SP coupling to predict memory recognition would be a function of prior encoding strength.

Finally, just as reactivation of newly encoded memory traces in the sleeping brain has been demonstrated to help us retain memories^25^, neural reactivation of encoding patterns during remembering also has been proven to facilitate retrieval^26^. However, evidence linking memory consolidation processes operating during sleep with subsequent reactivation of encoding activity patterns during retrieval is lacking. To address this question, we investigated the relationship between the degree of SO-SP coupling during the night following training and the extent of encoding-retrieval pattern similarity during successful memory recognition the next morning. Building on accumulated evidence that memory consolidation during sleep involves a gradual transformation and integration of representations in neocortical networks^27,28^, we hypothesized that more precise temporal coordination of SPs by SOs would be associated with decreased reactivation of encoding-related EEG patterns at retrieval.

## Materials and Methods

### Participants

Twenty-seven University students [age 21.8 ± 2.6 (mean ± SD), range 18–27 yr, 15 females] participated in the study. They had normal or corrected-to-normal vision, regular sleep habits confirmed by a structured interview and sleep-diaries over one week prior to participation in the experiment, and no history of neurological and/or psychiatric diseases. All participants gave informed and written consent to participate in the study. The experimental protocol in this study was reviewed, approved, and carried out according to the guidelines of the Ethical Committee for Human Research at the Pablo de Olavide University according to the principles outlined in the Declaration of Helsinki.

### Experimental paradigm

The experimental paradigm used in the present study has been described in detail elsewhere^29,30^. Task timing and stimulus delivery were controlled by Presentation® software (Neurobehavioral Systems, Inc.).

During the training session (Fig. 1A, left panel), participants were instructed to perform a semantic/perceptual-matching task, during which they were presented with 48 pairs of famous people’s faces of the same gender. Celebrities with the same profession were presented intermixed with celebrities of different profession in 8 consecutive blocks. Each pair was repeated 4 times in alternating blocks in a random fashion. Following the face pair presentation for 2 s, subjects were trained to maintain faces and their professions for 5 s while fixating on a cross in the center of the screen. Next, one face and one profession (probe stimuli) were presented for 3 s. Participants were then asked to respond by pressing the corresponding button on the response box (Cedrus®, model RB-530, Cedrus Corporation, San Pedro, CA, USA) whether the face and profession, on the left or right side, corresponded to the study face in that particular position. They were forced to give a different response in each repeated trial, which guaranteed that their attention was focused on the relevant information during the encoding and retention phases. Importantly, participants were informed that memory for face-face associations would be tested the following morning, since previous evidence suggests that sleep facilitates retention of associative memories based on relevance for future use^31–36^.

During the recognition task (Fig. 1A, right panel), all faces were presented both coupled with the same face as in the training phase (intact condition), and recombined with a different face (rearranged condition), while controlling that the rearrangement maintained the gender and semantic context (same or different profession) of the training phase. Each face appeared either in one condition or the other in two different blocks (24 pairs per block were intact and the other 24 rearranged). Participants were asked to respond as fast and accurately as possible as to whether or not the two faces had been presented together during the training phase.

### Experiment design and procedure

The experimental protocol is illustrated in Fig. 1B. All participants were trained in the evening (18:30 h), under conditions of either normal sleep duration (NSD; from 12:00 h to 8:00 h; N = 13) or acute sleep restriction (ASR; from 4:00 h to 8:00 h; N = 14) the night before. During the ASR session, participants were allowed to read and watch videos while a technician observed them to prevent them from sleeping. Participants were instructed to refrain from napping from the week prior to the first experimental session until the end of the experiment, aspect that was corroborated by sleep diaries.

Recognition memory for paired associates was tested the next morning after a full night’s sleep. We avoided the inclusion of a memory test after repeated encoding because retrieval practice has proven to be more effective than repeated study^37^ and equally effective as sleep^38^ for improving long-term memory. EEG recordings were collected the night before and after training, as well as during the training and recognition phase. Only the night following training was analyzed for the purpose of the present study.

### EEG acquisition

EEG data were collected from 59 scalp electrodes (Grass, USA) referenced to linked mastoids and positioned according to the extended International 10–20 system (Fig. S1). Additional electrodes were used to distinguish between vertical and horizontal eye movements and to monitor submental muscle tone. EEG recordings were amplified (BrainAmp MR, Brain Vision®), bandpass-filtered between 0.1–100 Hz, and sampled at 250 Hz.

### Behavioral data analysis

Subjective sleepiness levels were assessed just before starting the training task with the Epworth Sleepiness Scale^39^. Sustained attention with repeated presentation was also evaluated as in Alberca-Reina *et al*.^30^. Particularly, we analyzed false alarms in two or more consecutive trials, anticipations (reaction times -RT- shorter than 300 ms), long delays (RT longer than 2500 ms), and intra-subject variability of RT to correctly recognized face pairs calculated with the intra-individual coefficient of variation (iCV; the ratio between the intra-individual standard deviation and the individual mean). Finally, task performance was measured across repeated study by computing the mean RT for hits, as well as the hit rate and the false alarm rate.

Behavior during the recognition task was assessed on the basis of different indices including hit, correct rejection, miss, and false-alarm rates. The *d’* index was obtained by subtracting the z-score for the false-alarm rate from the z-score for the hit rate^40^.

### EEG preprocessing

Extracerebral artifacts were partially removed from EEG signals by applying independent component analysis (Infomax algorithm) as implemented in the BrainVision Analyzer software v. 1.05 (Brain ProductsV® GmbH). The remaining noisy EEG epochs were manually rejected by visual inspection. Artifact-free EEG epochs were transformed into the common average ref. ^41^, and band-pass filtered (0.5–30 Hz) using a finite impulse response filter with a Kaiser window (order = 1326). EEG data were then epoched into 1 s segments relative to onset presentation of each paired associate during both the training and recognition phase. For each participant, epoched trials were further classified as correctly remembered or forgotten during the recognition task. We originally planned to investigate whether semantic congruence (same *vs*. different profession) was a modulating factor of the main hypotheses of the study. Unfortunately, the number of available artifact-free EEG trials did not allow us to address this issue. Supplementary Table S1 shows the mean number (and standard deviation) of artifact-free EEG trials used to address group differences (based on remembered paired associates) and differences between subsequently remembered and forgotten paired associates for every repetition during training and retrieval. In order to make the remembered and forgotten conditions comparable in terms of signal-to-noise ratio, the number of remembered face-face pairs was matched with the number of forgotten pairs.

### Spatiotemporal EEG pattern similarity at encoding and retrieval

To determine the degree of similarity between EEG patterns, we adopted the spatiotemporal pattern similarity (STPS) approach developed by Lu and colleagues^13^. Spatiotemporal vectors for each paired associate of interest were constructed from the epoched EEG data depending on the tested hypothesis. For each single trial, the vector included the mean EEG voltage from one of the six regions as representative of spatial features (see Fig. S1) and a sliding window of 200 ms (50 time points) in time steps of one time point as representative of temporal features. Finally, the data were grouped into 20 ms bins, resulting in the 40 time points. The degree of EEG similarity between trials was calculated with Pearson’s correlation coefficients, which have shown to be insensitive to the absolute amplitude and variance of the EEG response. The correlation coefficients were then converted to Fisher’s *z* scores for subsequent statistical analyses.

This approach was specifically adapted to assess the following hypotheses. Firstly, we tested whether reinstatement of EEG activity patterns across repetitions of the 48 paired associates (content-specific STPS) during successful encoding was greater in the NSD group than in the ASR group, as previously reported following a night of total sleep deprivation^19^. Similarity analysis was applied to EEG patterns associated with the 1^st^ and 2^nd^ repetition, with the 2^nd^ and 3^rd^ repetition, and with the 3^rd^ and 4^th^ repetition of the same face-face pair across all paired associates. This was done for subsequently remembered and forgotten pairs, separately. Although our approach is equivalent to that proposed by Lu *et al*.^13^, results derived from each element of the diagonal matrix were not averaged. This procedure allowed us to determine the required number of repetitions for identifying group differences in the stability of EEG patterns with repeated study and the contribution of such stability to recognition memory.

Secondly, if the stability of neural representations during encoding is negatively affected by sleep restriction, the fidelity of reinstatement associated with successful encoding should be reduced in the ASR group in line with previous evidence^42^, unless consolidation during sleep has the potential to strengthen poorly encoded memories^3,5,7,8^. In the latter case, cortical reinstatement revealed by the similarity between the EEG activity elicited by the 4^th^ repetition of paired associates at encoding and the same paired associates presented at retrieval (i.e., intact face-face pairs) would be comparable between the two groups. Alternatively, if sleep promotes reorganization of new memory representations over distributed brain circuits^27,28^, the ASR group would show a lower degree of similarity between encoding and retrieval activity patterns as compared with the NSD group. This analysis was also performed for remembered and forgotten paired-associates, separately.

### Analysis of sleep structure

Experienced researchers manually scored sleep following training in individual 30-s epochs according to the guidelines of the American Academy of Sleep Medicine^43^. Total sleep time (TST), sleep onset latency (SOL), R (REM sleep) latency, and the duration and percentage of sleep stages (N1, N2, N3, R) based on the TST were determined. SOL was the time from lights out to the first epoch of stage N1, and R latency was the time from SOL to the first epoch of R sleep. Finally, sleep efficiency was also computed as the percentage of time sleeping as a function of the time in bed.

### Coupling between SPs and SOs

First, SOs were automatically identified over frontal and frontocentral sites (F3, F1, Fz, F2, F4, FC1, FC2, Cz) in stage N3 using a standard algorithm described elsewhere^44^. SPs were also identified in stage N3 over frontocentral (F7, F5, F3, F1, Fz, F2, F4, F6, F8, FT7, FC5, FC3, FC1, FCz, FC2, FC4, FC6, FT8) and centroparietal sites (TP7, CP5, CP3, CP1, CPz, CP2, CP4, CP6, TP8, P7, P5, P3, P1, Pz, P2, P4, P6, P8). We focused on fast SPs (13–16 Hz) because they have been more consistently related to memory consolidation than slow SPs (9–12 Hz)^45,46^, and have shown strong phase synchronization with the depolarizing up-state of SOs^21–24,47,48^. Further details about the identification of SOs and fast SPs can be found in Supplementary Methods S1.

The SO-SP coupling was determined using the approach developed by Mölle and colleagues^48^. Briefly, event correlation histograms of fast SPs were referenced to the negative half-wave peaks of the SOs using 6 s windows with 3 s offsets and a bin size of 48 ms. For SP counts, SP peaks and troughs of all detected SPs were computed from all EEG electrodes used for either frontocentral or centroparietal SPs identification. SP counts in each time bin were divided by the number of SOs, and then divided by the bin width to obtain the event rate per second (Hz). The resulting signal was baseline corrected after applying mean centering to each EEG electrode.

### Statistical analysis

We first evaluated whether behavioral indices and sleep parameters corresponding to the night following memory acquisition deviated from normality by applying the Kolmogorov-Smirnov test with the Lilliefors correction. Depending on whether or not normality could be assumed, group differences were evaluated by applying either the Student’s *t*-test or the Mann-Whitney *U* test, respectively. In all cases, Bonferroni correction was used to counteract the multiple testing problem.

Paired *t*-tests were applied to assess differences between STPS obtained during the training and recognition phase associated with remembered and forgotten paired-associates, while unpaired *t*-tests were used to evaluate group differences. We further used linear regression analysis to investigate whether SO-SP coupling during the night of recovery sleep was associated with both the stability of neural representations indexed by STPS across repetitions during training and the magnitude of encoding-retrieval STPS (STPS_E-R_) during the recognition task. To control for multiple testing, we applied a nonparametric statistical method based on cluster-level randomization testing (with 10,000 randomizations) that controls the family-wise error (FWE) rate^49^. This method is implemented in the FieldTrip toolbox (http://www.fieldtriptoolbox.org/). Further details about this procedure can be found in Supplementary Methods S2. For all results, we further report the effect size and its 95% confidence interval (*CI*_*0.95*_) based on bootstrapping resampling (for more details, see Supplementary Methods S3).

Finally, we applied one-level random intercept mixed-effects logistic regression models to evaluate whether the probability of correctly recognizing a face-face pair following a night of recovery sleep was a function of encoding strength (i.e., encoding STPS), mechanisms of active systems consolidation processes during sleep (i.e., SO-SP coupling), and reinstatement of encoding-related activity (STPS_E-R_). All non-binary predictor variables were standardized and mean-centered. A variance inflation factor greater than 5 was used as an indicator of multicollinearity^50^. We started with a model including one fixed effect and subjects as a random effect, and next continued adding fixed effects in a stepwise fashion. We selected the model with the lowest AIC value (Akaike information criterion). The regression coefficients were tested for significance with the Wald test^51^, and transformed to odds ratios (ORs) and their 95% confidence intervals (*CI*_*0.95*_) for reporting purposes. These analyses were conducted using the lme4 procedure implemented in R v3.0.1^52,53^.

## Results

### Effects of sleep restriction on recognition memory

Sleepiness and attention during training were not affected by the sleep manipulation applied in the night before (Supplementary Results S1 and Table S2).

During the recognition task, the ASR group showed a higher hit rate (0.68 ± 0.11) compared with the NSD group (0.59 ± 0.15). Although the difference did not reach statistical significance it cannot be completely discarded because the effect size was significant (*t*_*(25) = *_1.82, *p* = 0.08, *d* = 0.68, *CI* = [0.63 1.75], *CL* = 0.50). The same happened with the false alarm rate (NSD: 44.7 ± 19.0; ASR: 38.8 ± 10.9; *t*_*(25) = *_1.01, *p* = 0.32, *d* = 0.38, *CI* = [0.02 2.05], *CL* = 0.27), which likely contribute to explain why the two groups showed comparable memory accuracy (*d’*) during the recognition phase following a regular night of sleep (NSD: 0.61 ± 0.52; ASR: 0.53 ± 0.32; *t*_*(25) = *_0.53, *p* = 0.6, *d* = 0.2, *CI* = [−0.33 1.28], *CL* = 0.15).

### Effects of sleep restriction on encoding strength

**Figure 2:**
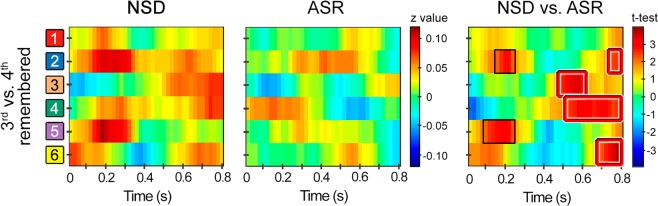
Effect of ASR on STPS across repeated study at encoding of subsequently remembered paired-associates. Within-subjects STPS, expressed as averaged *z*-values, between the encoding EEG activity patterns elicited by the 3^rd^ and 4^th^ repetition of subsequently remembered paired-associates in the recognition task performed by the NSD group and the ASR group. The x-axis represents time, and the y-axis the spatial locations shown in Fig. S1. The statistics of contrasting STPS between groups (NSD vs. ASR) is shown in the right panel. The red and black squares refer to significant clusters showing greater encoding STPS for the NSD group compared to the ASR group that either survived or not FWE correction, respectively.

The primary research question of the present study was to test whether sleep restriction impaired the memory strength of the novel encoded associations during the subsequent awake period. In line with this hypothesis, the ASR group of participants, when compared to the NDS group, showed a reduction of the stability of neural patterns during the repeated encoding of the same paired associates (Fig. 2). More concretely, encoding STPS was higher in the NSD group in two time windows at stimulus onset, an earlier one, at around 100–300 ms, over right frontal and left parietal regions did not survive FWE correction but the size effect based on the mean of the cluster was significant (*t*_*(25)*_ = 3.06, *p*_*uncorrected*_ = 0.005, *d* [*CI*_*0.95*_] = 1.16 [1.10 1.21], *CL* = 0.84). In addition, a later time window from stimulus onset, at around 500–800 ms, over the right frontal, right parietal, and bilateral central locations survived multiple correction testing (*t*_*(25)*_ = 3.46, *p*_*cluster-corrected*_ = 0.024, *d* [*CI*_*0.95*_] = 1.602 [1.601 1.603], *CL* = 1.16). Interestingly, only stability of neural patterns in the later time window was determinant of subsequent recognition memory (Supplementary Results S2 and Fig. S2 and S3).

### Relationship between encoding strength and SO-SP coupling

**Figure 3:**
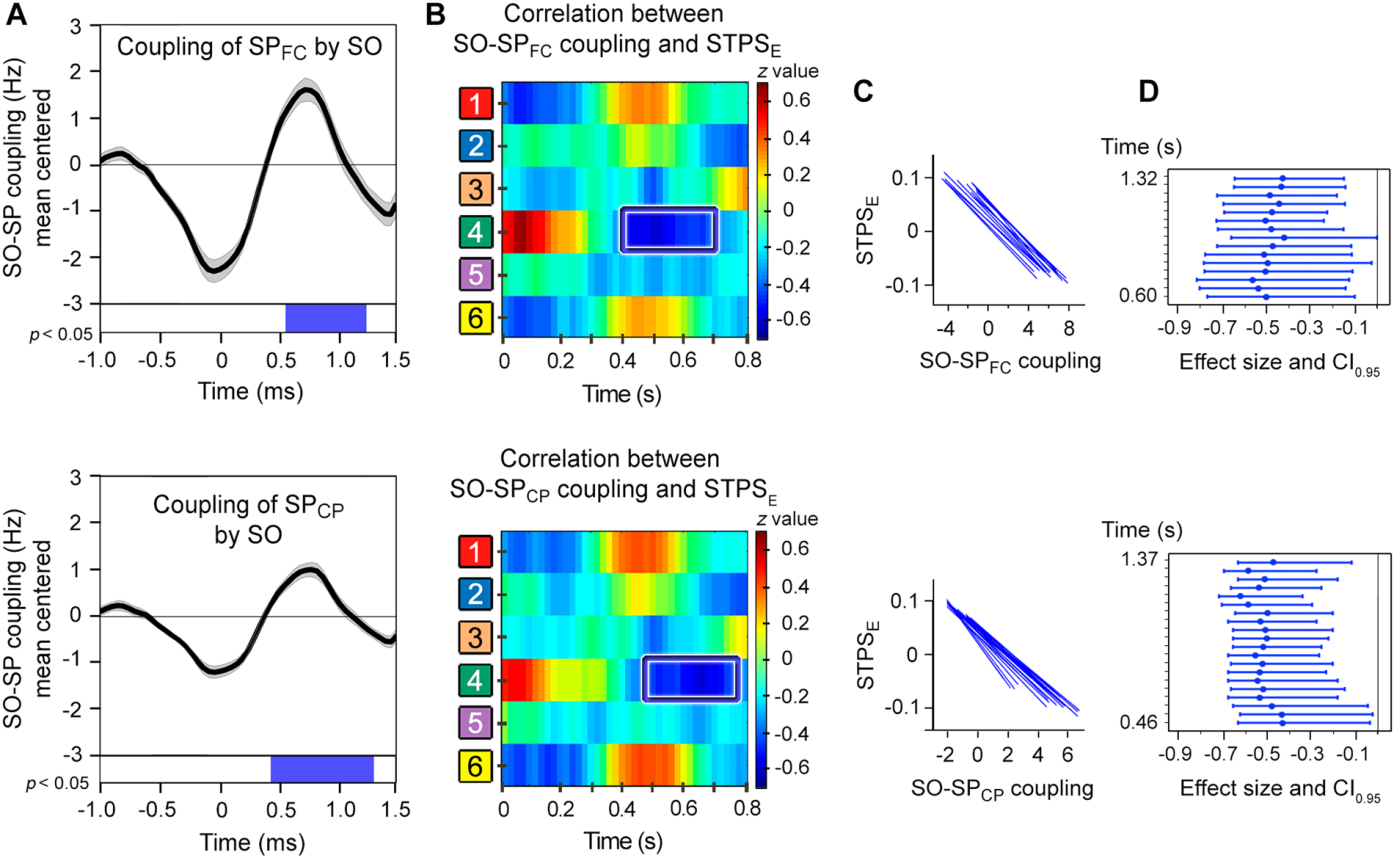
Contribution of encoding STPS to SO-SP coupling during SWS in the recovery sleep night. (**A**) Event correlation histogram between SO and fast SPs localized over frontocentral (top) and centroparietal electrodes (bottom) for all participants. The blue box indicates the time interval where SO-SP coupling was negatively correlated with the encoding STPS (*p*_*uncorrected*_ < 0.05). (**B**) *Z* transformation of Pearson correlation coefficients between SO-SP coupling and the encoding STPS (3^rd^ vs. 4^th^ repetition) for remembered paired-associates at one representative time point of the SO upstate (1.03 s for both frontocentral and centroparietal SPs). The blue square refers to the cluster showing negative correlation (*p*_*uncorrected*_ < 0.05). (**C**) Regression slopes of significant correlations for both frontocentral (top) and centroparietal SPs (bottom). (**D**) Effect sizes (Pearson’s *r*) and *CI*_*0.95*_ of significant correlations.

We next asked whether the stability of neural response patterns elicited by repeated encoding of the same stimuli was associated with the interplay of sleep oscillations associated to memory consolidation. No significant correlations were found between the encoding STPS and the suppression of fast SPs during the down-state of SOs. However, one temporal cluster emerged when these correlations were limited to the up-state interval, but only for paired associates that were successfully recognized (Fig. 3). These correlations were negative over right central regions between 400–600 ms for frontocentral SPs (−0.54 < *r*_*(25)*_ < −0.43, 0.004 < *p*_*uncorrected*_ < 0.026) and between 500–700 ms for centroparietal SPs (−0.53 < *r*_*(25)*_ < −0.42, 0.004 < *p*_*uncorrected*_ < 0.029). Although the clusters did not survive multiple correction testing, the effect sizes were statistically significant (frontocentral SPs: −1.06 < [*CI*_*0.95*_] < −0.03; centroparietal SPs: −1.09 < [*CI*_*0.95*_] < −0.01) and covered part of the cluster where the NSD group showed greater STPS for remembered events compared to the ASR group during late encoding.

In addition, we explored if the effects of sleep restriction were appreciated in the macrostructure and microstructure of sleep in the subsequent night, but no significant effects were found (Supplementary Results S3, Table S3, Table S4, and Fig. S4).

### Relationship between SO-SP coupling and reinstatement of encoding-related EEG patterns during retrieval

**Figure 4:**
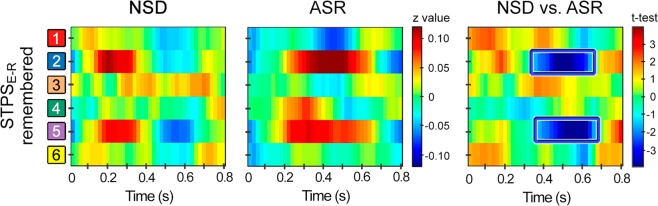
Effect of ASR on encoding-retrieval STPS for remembered paired-associates. Within-subjects STPS, expressed as averaged *z*-values, between the EEG activity patterns elicited by the 4^th^ repetition of paired associates at encoding and the same paired associates presented at retrieval for remembered associations in the recognition task performed by the NSD group and the ASR group one day after training. The x-axis represents time, and the y-axis the spatial locations shown in Fig. S1. The statistics of contrasting STPS between groups (NSD vs. ASR) are shown in the right panel. The blue squares refer to significant clusters where the ASR group showed greater encoding-retrieval STPS compared to the NSD group after applying FWE correction.

Reinstatement of encoding-related activity during retrieval (STPS_E-R_) was evaluated by estimating the STPS between the EEG activity associated with the 4^th^ presentation of paired associates at encoding and the EEG activity elicited by the same pairs presented at retrieval. The two groups showed a similar pattern of similarity between encoding and retrieval over right frontal and left posterior regions at approximately 150–350 ms after stimulus onset (Fig. 4). However, the ASR group reinstated this pattern of activity for a longer period, up to approximately 700 ms (Fig. 4, right panel). Indeed, the ASR group showed greater STPS_E-R_ than the NSD group over right anterior and left posterior regions from about 340 to 700 ms (*t*_*(25)*_ = −4.98, *p*_*cluster-corrected*_ = 0.007, *d* [*CI*_*0.95*_] = −1.752 [−1.750 −1.753], *CL* = 1.28). The standardized mean difference was of huge magnitude, so that the probability that a sleep-restricted individual showed higher STPS_E-R_ than a control participant was almost 90%. Importantly, the STPS_E-R_ also contributed in explaining variations in memory performance across all participants (Supplementary Results S4 and Fig. S5).

**Figure 5:**
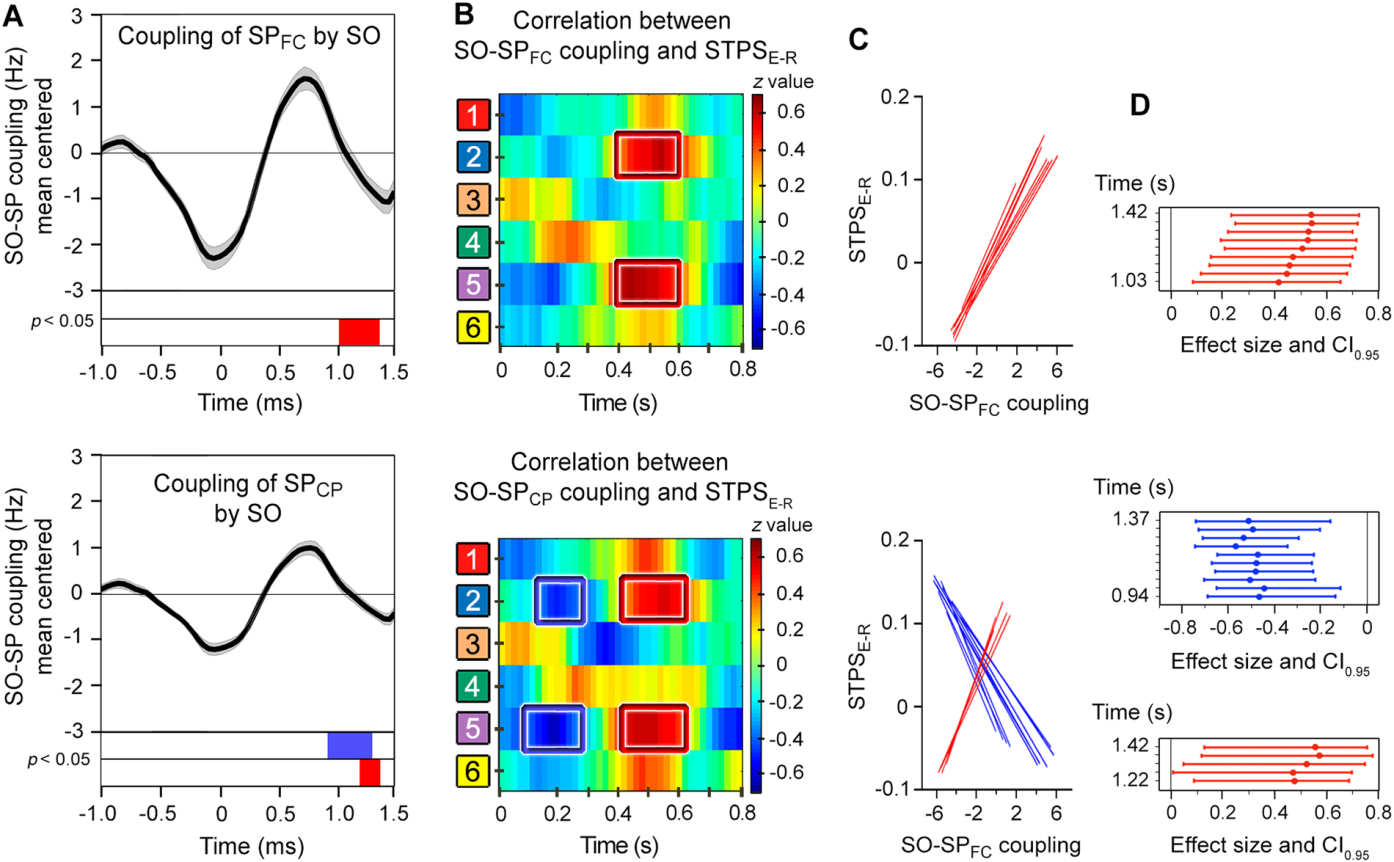
The relationship between SO-SP coupling during post-training sleep and encoding-retrieval STPS. (**A**) Event correlation histogram between frontocentral SOs and fast SPs localized over frontocentral (top) and centroparietal electrodes (bottom) averaged across all participants. The blue and red boxes indicate the time intervals where SO-SP coupling was negatively and positively correlated with the encoding-retrieval STPS, respectively. Note that correlations were significant in the up-state-to-down-state transition, although they did not survive multiple testing (*p*_*uncorrected*_ < 0.05). (**B**) Pearson correlation coefficients between SO-SP coupling and the encoding-retrieval STPS for remembered paired-associates at one representative time point of the SO up-state (1.32 s for both frontocentral and centroparietal SPs). The blue and red squares refer to clusters showing significant negative and positive correlations that did not survive FWE correction (*p*_*uncorrected*_ < 0.05), respectively. (**C**) Regression slopes of significant correlations for both frontocentral (top) and centroparietal SPs (bottom). (**D**) Effect sizes (Pearson’s *r*) and *CI*_*0.95*_ of significant correlations.

We next sought to address the third main question of the present study, whether the degree of SO-SP coupling during the night of recovery sleep was associated to memory reinstatement during retrieval in the day after. Results showed that the SO-SP coupling across participants, particularly in the upstate-to-downstate transition, was associated to neural similarity measured between remembered events (but not between forgotten ones) over right frontal and left parietal regions in two different time intervals (Fig. 5A–C). Specifically, we found negative correlations at 100–300 ms for centroparietal SPs (−0.57 < *r*_*(25)*_ < −0.43, 0.023 < *p*_*uncorrected*_ < 0.001) but positive correlations at 400–600 ms for both frontocentral (0.44 < *r*_*(25)*_ < 0.60, 0.02 < *p*_*uncorrected*_ < 0.0007) and centroparietal SPs (0.48 < *r*_*(25)*_ < 0.60, 0.1 < *p*_*uncorrected*_ < 0.001). Although these results did not survive FWE correction, the effect sizes shown in Fig. 5D were statistically significant (frontocentral SPs: 0.09 < [*CI*_*0.95*_] < 0.76; centroparietal SPs for negative cluster: −0.73 < [*CI*_*0.95*_] < −0.11; centroparietal SPs for positive cluster: 0.01 < [*CI*_*0.95*_] < 0.13).

### Contribution of encoding strength, SO-SP coupling and reinstatement of encoding processes during retrieval to recognition memory

Finally, we wanted to know the extent to which the probability of correct recognition was influenced by the interaction of encoding strength with the SO-SP coupling during the recovery night, and with the reinstatement of encoding-related activity during retrieval. To assess this issue, we implemented a mixed-effects logistic regression analysis. This approach allows introducing random effects to capture variation across subjects. More concretely, recognition memory was modeled as a binary outcome with (i) **encoding STPS** (i.e., the mean of the cluster where encoding STPS was higher in the NSD group than in the ASR group), (ii) **temporal grouping of SPs by the up-state of SOs**, (iii) **STPS**_**E-R**_ either in the early or late time windows where the two groups showed similar and different degree of cortical reinstatement respectively, and (iv) **interaction terms** as fixed effects (model including early STPS_E-R_: N = 1620, AIC = 1591.8, log-likelihood = −785.9; model including late STPS_E-R_: N = 1620, AIC = 1586.9, log-likelihood = −784.4). Importantly, the variance inflation factor for all predictors in the two models, including interaction terms, were below 1.52, which indicates low correlation between variables.

Results showed that the probability of correct recognition was predicted by increased encoding STPS (*OR* = 2.56 [1.85 2.75], *p* < 10^–16^), increased early STPS_E-R_ (*OR* = 7.79 [6.16 9.84], *p* < 10^−16^) and increased late STPS_E-R_ (*OR* = 5.21 [4.12 6.57], *p* < 10^−16^). The degree of SO-SP coupling also enhanced the probability of correctly recognizing a face-face pair, but this association was moderated by prior encoding strength and reinstatement during retrieval as suggested by the three-way interactions. Accordingly, under conditions of weak encoding, as in the ASR group, the SO-SP coupling enhanced the probability of correct recognition if the degree of reinstatement of encoding-related activity was high at either early (*OR* = 0.73 [0.55 0.96], *p* = 0.02) or late time intervals (*OR* = 1.60 [1.08 2.39], *p* = 0.02). But if encoding of memory was strong, as in the NSD group, reinstatement of previous encoding processes contributed to increase the probability of correct recognition regardless of SO-SP coupling strength.

## Discussion

The study revealed that sleep restriction disturbs the encoding of new information without impairing reinstatement of encoding-related activity patterns or recognition memory evaluated after a full night’s sleep. This paradox might be accounted for by the activation of specific neural mechanisms of memory consolidation during sleep. Our findings showed that sleep restriction decreased the stability of neural patterns throughout the repeated encoding of stimuli during the subsequent awake period. The decrease of encoding strength was associated with enhanced temporal grouping of fast SPs by the SO during the following night of recovery sleep; and the increased SO-SP coupling was, in turn, associated with the reinstatement of encoding processes during retrieval the next morning. Importantly, the degree of SO-SP coupling emerged as an important determinant of successful recognition only for memories that were weakly encoded during the previous awake time. The current study provides novel insights into the dynamic interplay between awake and sleep memory processes.

### Sleep restriction reduces encoding strength

Here, we provide novel evidence that restriction of sleep time to 4 h the night before training by applying a bedtime delay is sufficient to disrupt the process of memory stabilization underlying a repeated exposure to the same event^16^. Using pattern similarity analysis, we found that multiple encodings of the same event elicited less stable EEG patterns in sleep-restricted participants relative to participants who obtained a full night of sleep before training.

It is unlikely that the negative impact of sleep restriction on encoding strength was caused by a decrease in the level of alertness or sustained attention. Indeed, the two groups of participants showed similar levels of sleepiness, comparable performance in the training task, and a similar degree of variability in RT across trials, confirming results from previous studies^1,54^. However, the influence of top-down control of sensory processing, as suggested by previous results^18,19,55^, cannot be discarded, because the stability of EEG patterns was also reduced during early encoding over frontoparietal regions after a shortened night of sleep.

Additionally, the adverse effects of sleep restriction on the stability of neural representations could have been mediated by changes in the activation level of the hippocampus during encoding, which is required to build distinct, pattern-separated representations^56,57^. In support of this idea, encoding hippocampal activity has been found to be significantly reduced following a night of sleep disruption^1^ or total sleep deprivation^2^, and to be predictive of the amount of recovered information^58^ and of the magnitude of cortical reinstatement during retrieval^42,59^. These results are consistent with the fact that both encoding strength and reinstatement of previous encoded-related neural patterns emerged in the present study as key determining factors of successful recognition.

### Sleep promotes strengthening of weakly encoded memories

Sleep restriction, like total sleep deprivation^1,18,19^ or SWS disruption^2^, disturbed encoding of new information, but without impairing memory retrieval following one night of recovery sleep. Taken together, these results suggest that our sleep manipulation likely produced less severe effects on encoding processes, thereby allowing the brain to restore weak memories during subsequent sleep. Studies applying targeted memory reactivation, a method for cueing the reactivation of specific memories in SWS, suggest that this procedure mainly facilitates consolidation of those memories that were recalled with a low degree of accuracy prior to sleep^3,4^. Supporting these results, the strength of encoded memories in the present study was negatively related to the temporal grouping of fast SPs by the SO up-state during the subsequent night’s sleep, so that the weaker the encoding, the higher the coupling strength between SPs and SOs. But the most striking result was that the interplay between brain oscillations during SWS only contributed to predict correct recognition for memories that were poorly encoded during the day before. These findings strongly suggest that the temporal dynamics between SPs and SOs in consolidating hippocampus-dependent memory^48,60–66^ is modulated by the strength of prior encoding.

According to the model of active system consolidation during sleep, recently encoded memories that are reactivated during subsequent sleep have a greater likelihood to undergo qualitative changes, making them stronger to interference and more resistant to forgetting^25,27,28,67^. If the reactivation of prior memories during SWS, indexed here by the degree of SO-SP coupling, leads to a transformation or elaboration of the memory representations, retrieval should be less dependent on the reinstatement of certain encoding processes^68^, especially those referred to reactivation of nonessential information. In line with this prediction, SO-SP coupling correlated negatively with the reinstatement of frontoparietal neural pattern of activity elicited at early stages of encoding. In contrast, sustained reinstatement of the frontoparietal pattern across time was greater with the increased coupling between SPs and SOs in the previous night. These two apparent paradoxical results could be reconciled if we consider the possibility that distinct processes are being reactivated during early and late retrieval: one more focused on perceptual or contextual aspects of the event, and the other more related to later stages of memory retrieval involving, for example, semantic reactivation or executive control operations to regulate the attention towards the reactivated memories.

## Conclusions

Late bedtime habits are, unfortunately, a common practice among young adults. The current study shows that insufficient nocturnal sleep has a direct impact on learning mechanisms during the subsequent awake period, leading to weak memory formation for novel events. Weakly formed memories however, have a higher capacity to interact with sleep neural mechanisms underlying memory consolidation during a recovery night sleep, which in turn, helps restore memory representations to be accessible during later awake retrieval. Altogether, these findings support the notion that sleep may promote the strengthening of weakly encoded memories due to reduced sleep time in the night before.

## Supplementary information

SUPPLEMENTARY MATERIAL

## Data Availability

The datasets generated during and/or analyzed during the current study are available from the corresponding author on reasonable request.
