# Supplementary material for: Weakly encoded memories due to acute sleep restriction can be rescued after one night of recovery sleep
Source: Sci Rep. 2020 Jan 29;10:1449. doi: 10.1038/s41598-020-58496-4 (PMC6989495; doi:10.1038/s41598-020-58496-4)
Supplement: Supplementary file 1 — SUPPLEMENTARY MATERIAL [file 41598_2020_58496_MOESM1_ESM.pdf]

SUPPLEMENTARY MATERIAL

Weakly encoded memories due to acute sleep restriction can be rescued after one night of recovery sleep

Daniel Baena, Jose L. Cantero, Lluís Fuentemilla, Mercedes Atienza

Supplementary Methods

Methods S1. Identification of SOs and SPs

Identification of slow oscillations

First, the EEG data was band-pass filtered between 0.5 and 3.5 Hz using a finite impulse response (FIR) filter. Next, time points of positive to negative zero crossings were computed, and the lowest and highest values between every 2 of these time points were detected. SOs were defined as intervals between consecutive positive-to-negative zero crossings with a length of 0.9 to 2 s if the corresponding negative peak amplitude was lower than −80 μV and the corresponding amplitude difference (positive peak minus negative peak) was ≥ 140 μV. Averages of original EEG data in a 3 s window ± 1.5 s around the peak of the negative half-wave of all detected SOs were computed. We calculated SO number, density (number per minute), mean duration, and mean peak-to-peak amplitude, as well as the duration of the down-states and up-states at each EEG electrode. These indices were finally averaged across EEG electrodes for each participant.

Identification of sleep spindles

We first performed a manual identification of sleep SPs in EEG electrodes selected for visual inspection (F3, F4, C3, Cz, C4, P3, Pz, P4, O1, O2). Although recent research has focused on providing a standard to define sleep SPs (Mei et al., 2017; O'Reilly et al., 2017; Purcell et al., 2017; Ray et al., 2015), the definition of these events varies across studies, which likely contributes to the difficulty of their automatic classification. Based on previous data comparing automated SP detection with expert manual SP scoring (Ray et al., 2015), start and end markers were placed in the EEG derivation that showed maximum amplitude if the SP fulfilled the following criteria: (i) duration

varying between 0.5 and 3 s; (ii) minimum amplitude of 15 μV; (iii) fusiform morphology; and (iv) minimum inter-spindle interval of 1 s (Urakami et al., 2012).

Next, we applied an automatic sleep SP analysis based on an established algorithm previously described (Mölle et al., 2011). To reduce the number of false positives, the automatic identification was restricted to the time window established by manual markers. Although fast SPs are more evident in centroparietal electrodes as opposed to the more frontocentral expression of slow SPs (Andrillon et al., 2011; Cox et al., 2017; Werth et al., 1997; Zeitlhofer et al., 1997), recent evidence has shown that fast SPs are modulated by the SOs even at sites where they are less prominent (Cox et al., 2018), and that not only centroparietal but also frontocentral fast SPs can be a key determinant of offline memory consolidation for both motor learning (Mander et al., 2017) and episodic memory (Cairney et al., 2018). Building on this evidence, the automatic identification was then run at anterior and posterior electrodes separately. The former included frontal, frontocentral and frontotemporal EEG locations (F7, F5, F3, F1, Fz, F2, F4, F6, F8, FT7, FC5, FC3, FC1, FCz, FC2, FC4, FC6, FT8), while the latter included centroparietal, parietal and parietotemporal EEG locations (TP7, CP5, CP3, CP1, CPz, CP2, CP4, CP6, TP8, P7, P5, P3, P1, Pz, P2, P4, P6, P8).

Before applying the automatic algorithm, the EEG signal was band-pass filtered with a FIR between 13 and 16 Hz. Then, the root mean square (RMS) of the filtered signal was calculated at each sample point using a moving window of 0.2 s. The resulting RMS signal was smoothed with a moving average of 0.2 s. The threshold for SP detection in the RMS signal was set to 1.5 standard deviations of the filtered signal within the time interval established by the manual markers, as defined by the mean across EEG electrodes. A SP was detected when the RMS signal remained above the threshold for 0.5-3 s, and the beginning and end of the SP were marked at the threshold crossing points. For every detected SP, the peak and trough were defined as the maximum and minimum of the filtered signal (between the beginning and end of the SP) and the deepest trough was designated as the "SP peak" that represented the respective SP in time, i.e., the time point taken for referencing event correlation histograms. SP density, amplitude, duration, intensity (duration/amplitude) and dominant frequency was calculated for each EEG derivation.

Methods S2. Statistical analysis of STPS

For the STPS analysis, statistics were computed for every time point, and the time points whose statistical values were above a threshold (p = 0.05) were selected and clustered into connected sets on the basis of temporal and spatial adjacency. The cluster-level statistics were calculated by taking the sum of the statistical values within a cluster (e.g., t- or z-statistic). After applying a primary threshold to voxels, p-values (α = 0.05) were assessed by means of 10,000 permutations using the Monte Carlo method implemented in the Fieldtrip toolbox. Next, the maximum cluster statistics over all six regions were chosen to construct a distribution of the cluster-level statistics under the null hypothesis. The nonparametric statistical test was obtained by calculating the proportion of randomized test statistics that exceeded the observed cluster-level statistics.

Methods S3. Estimation of effect size and confidence intervals

To estimate the standardized effect size for the mean difference between groups, we first computed Cohen's d (Cohen, 1992) by dividing the mean difference by the pooled standard deviation and next applied the Hedges correction (Hedges' d) (Cumming, 2012) for small samples. Although Cohen classified standardized effect sizes as small (d = 0.2), medium (d = 0.5), and large ($d \geq 0.8$), these values are arbitrary. In order to facilitate their interpretation, we converted these standardized effect sizes into a percentage by calculating the common language (CL) (McGraw & Wong, 1992). It expresses the probability that an individual from one group (or treatment) has a higher value on one measurement than a person from the other group (or as compared to another condition). To estimate the standardized effect size between two mean dependent samples, we first computed Cohen's d_{rm}, and next applied the Hedges correction (Hedges' g_m) (Lakens, 2013). For correlation analyses, we report the correlation coefficient r as a standardized measure of effect size. In those cases in which a mixed ANOVA was applied, the partial eta squared (η^2_p) was computed as a measure of the effect size for the within-subjects factor. This index expresses the sum of squares of the effect in relation to the sum of squares of the effect plus the sum of squares of the error associated with the effect (Keppel, 1991).

Supplementary Results

Results S1. Effects of sleep restriction on behavioral performance during training

The NSD group (1.85 ± 0.55) and ASR group (1.57 ± 0.51) showed similar scores on the Epworth Sleepiness Scale during the training session ($t_{(25)}$ = 1.34, p = 0.19, d $[CI_{0.95}]$ = 0.51 [-0.09 0.66], CL = 0.37). Although this result was unexpected, the arousal level associated with the task instructions and the subjective nature of the sleepiness measure may partially account for the lack of group differences (Belenky et al., 2003; Leproult et al., 2003).

During the training phase, there were no statistically significant differences between groups in false alarms, anticipated responses (i.e, RT shorter than 300ms), long delays (i.e., RT longer than 2500ms), or intra-subject variability of RT (see supplementary Table S2). The mixed ANOVA only revealed an improvement of the hit rate ($F_{3,75}$ = 8.68, p = 0.0001, η^2_p = 0.26) and decreases of RT across repetitions ($F_{3,75}$ = 31.47, $p < 10^{-12}$, η^2_p = 0.56) that were independent of whether participants slept 4 or 8 h the night before (hit rate: $F_{3,75}$ = 1.28, p < 0.29, η^2_p = 0.049; RT: $F_{3,75}$ = 0.23, p < 0.87, η^2_p = 0.009).

Results S2. Contribution of encoding STPS to recognition memory

We evaluated whether the stability of EEG activity patterns during training, revealed by the strength of content-specific STPS across repetitions of face-face pairs, contributed to recognition memory. In particular, we compared the encoding STPS for subsequently remembered and forgotten events associated with the 1st vs. 2nd, 2nd vs. 3rd, and 3rd vs. 4th repetition.

The first contrast yielded greater STPS between the 1st and 2nd repetition of subsequently remembered paired-associates compared to subsequently forgotten paired-associates over central areas between 340 and 460 ms ($t_{(25)}$ = 5.79, $p_{cluster\text{-}corrected}$ = 0.038, d $[CI0.95]$ = 1.25 [0.70 1.79], CL = 0.91). Participants also showed greater STPS for subsequently forgotten than remembered associations in two temporal clusters. The former extended over the central areas from approximately 60 to 200 ms ($t_{(25)}$ = -6.12, $p_{cluster\text{-}corrected}$ = 0.001, d $[CI0.95]$ = -1.71 [- 2.38 -1.15], CL = 1.22) while the later was

evident over frontoparietal areas from 320 to 760 ms ($t_{(25)}$ = -5.84, $p_{cluster\text{-}corrected}$ = 0.027, *d [CI0.95]* = -1.46 [- 2.07 -0.78], *CL* = 1.03).

The second contrast (i.e., STPS between the 2nd and 3rd repetition) also revealed one early cluster associated with successful recognition over frontocentral areas between 220 and 440 ms ($t_{(25)}$ = 3.58 $p_{uncorrected}$ = 0.001, *d [CI0.95]* = 0.95 [0.43 1.51], *CL* = 0.68), and another cluster associated with forgetfulness that extended over the entire scalp between 300 and 800 ms ($t_{(25)}$ = -6.83, $p_{cluster\text{-}corrected}$ = 2 x 10^{-4}, *d [CI0.95]* = -2.10 [- 3.03 -1.02], *CL* = 1.51).

Interestingly, after the 4th repetition of the event (Supplementary Fig. S2), the stability of the EEG activity patterns that contributed to successful recognition memory emerged at the end of the interval, between 500 and 800 ms ($t_{(25)}$ = 5.83, $p_{cluster\text{-}corrected}$ = 0.002, d $[CI_{0.95}]$ = 1.07 [0.63 1.50], *CL* = 0.77), while the one contributing to forgetting was evident in an earlier time interval ranging from approximately 200 to 600 ms ($t_{(25)}$ = -6.51, $p_{cluster\text{-}corrected}$ = 8 × 10^{-5}, d $[CI_{0.95}]$ = -1.52 [-2.36 -0.75], *CL* = 1.07). Both clusters extended practically over the entire scalp. As illustrated in Fig. S2, the NSD and ASR groups showed similar patterns of EEG reactivation associated with memory recognition.

The stability of EEG activity patterns in response to the 4th repetition with respect to the previous presentation indicates that too much early reactivation of neural activity associated with previously encoded stimuli might be detrimental to long-term memory while the later-in-time reactivation might be beneficial; likely because a different kind of information is being reactivated at each moment. Consistent with this interpretation, Zhang et al. (2018) found that subsequent memory was only accounted for by stimulus-specific activity occurring between 500 and 1200 ms, but not by the neural activity occurring earlier in time. As predicted by the levels of processing framework (Craik & Lockhart, 1972), it is likely that differences observed in the late time interval reflect the contribution of deep semantic processing to more elaborate, longer-lasting, and stronger memory traces than the ones that would be produced by shallow levels of processing in earlier time intervals.

Additionally, we found that although the encoding STPS associated with the difference due to memory in the early and late time intervals were tightly related to each other

across all participants ($F_{1,31}$ = 30.2, p = 0.000005; for more details see Results S2 in supplementary material), only the STPS associated with remembered events in the late time window (Fig. S2D) was positively correlated across all participants with the index of recognition accuracy *d'* over frontal and right parietooccipital areas ($r_{(25)}$ $[CI_{0.95}]$ = 0.61 [0.27 0.81], $p_{cluster\text{-}corrected}$ = 0.02, CL = 1.13). This relationship was mainly evident in the NSD group ($r_{(11)}$ $[CI_{0.95}]$ = 0.74 [0.34 1.18], $p_{cluster\text{-}corrected}$ = 0.037, = CL = 0.96; Fig. S2E-F), but the comparison of regression coefficients and slopes did not reveal between group differences (Fig. S3A). The false alarm rate was the main determining factor of this association (Fig. S3B). Accordingly, the STPS associated with the difference due to memory was positively correlated with the false alarm rate (but not with the hit rate) within the same spatiotemporal window. The cluster did not survive FWE correction but the size effect was significant ($r_{(25)}$ $[CI_{0.95}]$ = -0.43 [-0.92 -0.12], $p_{uncorrected}$= 0.14, CL = 1.67). The same analyses were performed for the 1st vs. 2nd and for the 2nd vs. 3rd repetition, but the stability of EEG activity patterns across repeated study only contributed to improve recognition accuracy after the 4th presentation of face-face pairs.

Results S3. Effects of ASR on macrostructure and microstructure of sleep

Table S4 shows the polysomnography-derived sleep parameters in the two groups, and Table S5 the parameters defining SOs and fast SPs. No group differences were found for any parameter related to macrostructure of sleep after applying Bonferroni correction. The lack of group differences in N3% is consistent with results of a previous study in which the percentage of SWS recovered to baseline levels after four consecutive nights of 3 h of sleep restriction (Wu et al., 2010). The parameters defining SOs and fast SPs neither survived Bonferroni correction, with the exception of the duration of the depolarizing SO up-state. Differences in the up-state duration are particularly interesting because previous studies have related the length of the up-state with memory improvement (Heib et al., 2013). The authors of this study concluded that the longer duration of the depolarizing component of SOs might provide a longer time window for replaying and transferring the recently encoded memories to longer-lasting storage. On the contrary, recognition accuracy was not related to this parameter in our study; this was the case when all participants were combined ($r_{(25)}$ $[CI_{0.95}]$ = 0.37 [-0.13 0.74], CL = 0.62), and also when the analysis was performed on each group separately (NSD: $r_{(11)}$ $[CI_{0.95}]$ = 0.54 [-0.34 0.89], CL = 0.68; ASR: $r_{(12)}$ $[CI_{0.95}]$ = 0.09 [-0.42

0.60], CL = 0.52). The fact that we could not compute changes in memory performance from pre to post sleep may account for this discrepancy.

Next, we tested group differences in the SO-SP coupling. As previously reported (Mölle et al., 2002, 2011; Fogel & Smith, 2011; Staresina et al., 2015), event correlation histograms revealed a well-defined temporal relationship between fast SPs and SOs in both groups. Fig. S4A shows the temporal grouping of both frontocentral and centroparietal fast SPs by SOs in each group. With reference to the negative half-wave peak of the SOs, fast SP counts were suppressed around the down-state and increased during the up-state in the two groups. Although the ASR group showed a higher temporal coordination of fast SPs by SO up-states as compared to the NSD group (Fig. S4A), permutation testing did not reveal significant differences between groups, likely motivated by the small sample size. Accordingly, the effect sizes reached statistical significance for most time points in the SO up-state interval but not in the down-state period (Fig. S4B-C), regardless of whether SPs were detected over frontocentral (effect sizes ranged from 0.35 to 0.65) or centroparietal electrodes (effect sizes ranged from 0.38 to 0.88).

Results S4. Contribution of $\mathrm{STPS}_{\mathrm{E\text{-}R}}$ to memory performance

As illustrated in Fig. S5A, the $\mathrm{STPS}_{\mathrm{E\text{-}R}}$ over the first 150 ms over right frontal and bilateral parietooccipital areas was mainly associated with forgotten events ($t_{(25)}$ = -4.86, $p_{cluster\text{-}corrected}$ = 0.02, d_{rm} $[CI_{0.95}]$ = -0.86 [-1.19 -0.54], CL = 0.63), while reinstatement of both frontoparietal EEG-activity patterns from 200 to 450 ms ($t_{(25)}$ = 7.62, $p_{cluster\text{-}corrected}$ = 0.004, d_{rm} $[CI_{0.95}]$ = 1.15 [0.74 1.54], CL = 0.82) and frontocentral patterns between 520-800 ms ($t_{(25)}$ = 11.04, $p_{cluster\text{-}corrected}$ = 0.01, d_{rm} $[CI_{0.95}]$ = 0.93 [0.26 1.50], CL = 0.66) mainly contributed to successful recognition memory. While early reinstatement (i.e., 200-400 ms) associated with successful recognition was quite similar in the two groups, early reinstatement (i.e., first 500 ms) associated with recognition failure and late reinstatement (i.e., 400-600 ms) associated with successful recognition was mainly evident in the ASR group (Fig. S5B-C).

Supplementary Tables

Table S1. Artifact-free trials included in the analyses.

**Contrast**	NSD	ASR
**NSD vs. ASR**<br>(remembered paired-associates)		
STPS 1st vs. 2nd (training)	17.7 ± 6.5	20.1 ± 6.2
STPS 2nd vs. 3rd (training)	17.1 ± 6.2	19.0 ± 6.6
STPS 3rd vs. 4th (training)	22.6 ± 6.1	21.9 ± 5.1
$STPS_{E-R}$	23.9 ± 7.1	24.8 ± 5.6
**Remembered vs. forgotten**<br>(equal number of trials for both conditions)		
STPS 1st vs. 2nd (training)	12.6 ± 3.6	11.1 ± 4.0
STPS 2nd vs. 3rd (training)	12.4 ± 3.7	10.5 ± 3.4
STPS 3rd vs. 4th (training)	11.5 ± 4.1	11.9 ± 3.9
$STPS_{E-R}$	11.8 ± 4.6	11.6 ± 3.9

Results are expressed as mean ± SD (standard deviation).

Table S2. Behavioral indices of sustained attention per group in the training phase.

Indices	NSD	ASR	*p*	Hedges' *d* $(CI_{0.95})$	*CL*
FA	5.92 ± 5.07	4.36 ± 3.65	.36	0.34 (-0.09 1.77)	0.25
Anticipations	0.23 ± 0.44	1.07 ± 1.73	.10	-0.63 (-1.25 -0.58)	0.47
Long delays	3.54 ± 4.56	3.43 ± 3.86	.95	0.02 (-0.70 0.90)	0.01
iCV	0.24 ± 0.03	0.23 ± 0.04	.39	0.32 (-0.10 1.75)	0.24

Results are expressed as mean ± SD (standard deviation). FA = False alarms in two or more consecutive trials; iCV = intra-individual coefficient of variation; CL = common language

Table S3. Post-training sleep parameters in the NSD and ASR group.

**Sleep parameters**	**NSD (N013)**	**ASR (N = 14)**	$t_{(25)}$	*p*	***Hedges' d*** $(CI_{0.95})$	***CL***
TST (min)	478.81 ± 19.05	478.51 ± 10.26	0.50	.960	0.02 (-0.84 0.71)	0.01
SOL (min)	8.90 ± 6.41	5.22 ± 4.54	1.73	.095	0.65 (0.52 2.03)	0.47
N3 latency (min)	25.67 ± 11.79	16.89 ± 6.86	2.39	.025	0.89 (-4.70 3.58)	0.64
REM latency (min)	75.70 ± 29.59	81.51 ± 20.93	-0.59	.559	-0.22 (-1.02 0.39)	0.16
Wake (%)	3.52 ± 2.14	3.01 ± 1.12	0.78	.445	0.29 (-0.16 1.58)	0.21
N1 (%)	5.84 ± 2.54	3.47 ± 1.60	2.93	.007	1.09 (-0.75 2.95)	0.79
N2 (%)	37.53 ± 4.92	37.64 ± 7.43	-0.05	.963	-0.02 (-0.72 0.82)	0.01
N3 (%)*	27.23	29.71	65	.220	-2.23 (-7.33 1.36)	0.36
REM (%)	25.76 ± 4.96	25.50 ± 3.71	0.15	.879	0.06 (-0.64 0.89)	0.04
SE (%)	96.54 ± 2.02	96.97 ± 1.21	-0.61	.545	-0.23 (-1.48 0.27)	0.17

Results are expressed as mean ± SD (standard deviation). TST = total sleep time; SOL = sleep onset latency; SE = sleep efficiency. *These data show a significant deviation from normality, so the median, the Mann-Whitney U statistic, and the median difference are reported.

Table S4. Parameters defining slow oscillations and fast sleep-spindles in frontocentral and centroparietal locations during post-training sleep in the NSD and ASR group.

Parameters	NSD	ASR	$t_{(25)}$	*p*	Hedges' *d* ($CI_{0.95}$)	*CL*
**Slow oscillations**						
Dominant frequency (Hz)	0.85 ± 0.03	0.88 ± 0.03	-1.90	.069	-0.76 (-1.45 -0.74)*	0.55
Density (number/minute)	4.71 ± 2.70	3.97 ± 1.79	0.88	.393	0.32 (-0.07 1.59)	0.23
Amplitude (μV)	210.9 ± 36.2	204.7 ± 14.3	0.59	.558	0.22 (-0.3 1.42)	0.16
Duration (s)	1.21 ± 0.05	1.18 ± 0.04	2.08	.048	0.74 (0.72 1.47)*	0.54
Down state duration (s)	0.53 ± 0.04	0.52 ± 0.04	0.54	.593	0.21 (-0.33 1.29)	0.15
Upstate duration (s)	0.68 ± 0.02	0.66 ± 0.02	3.27	.003*	1.218 (1.217 1.219)*	0.88
**Fast frontal spindles**						
Dominant frequency (Hz)	13.70 ± 0.12	13.68 ± 0.11	0.45	.652	0.17 (-0.43 1.11)	0.12
Density (number/minute)	0.57 ± 0.33	0.55 ± 0.32	0.22	.828	0.08 (-0.61 0.89)	0.06
Amplitude (μV)	8.30 ± 2.45	8.29 ± 2.13	0.02	.987	0.01 (-0.74 0.78)	0
Duration (s)	0.82 ± 0.07	0.86 ± 0.08	-1.56	.130	-0.59 (-1.42 -0.43)*	0.43
Intensity (duration/amplitude)	0.11 ± 0.03	0.11 ± 0.03	-0.26	.793	-0.1 (-0.97 0.55)	0.07
**Fast parietal spindles**						
Dominant frequency (Hz)	13.7 ± 0.17	13.7 ± 0.14	0.28	.780	0.01 (-0.56 1.03)	0.07
Density (number/minute)	0.31 ± 0.21	0.33 ± 0.20	-0.16	.872	-0.07 (-0.85 0.67)	0.05
Amplitude (μV)	10.27 ± 3.18	9.55 ± 2.00	0.70	.488	0.26 (-0.20 1.61)	0.19
Duration (s)	0.82 ± 0.08	0.87 ± 0.08	-1.85	.077	-0.68 (-1.61 -0.61)*	0.49
Intensity	0.09 ± 0.03	0.09 ± 0.02	-0.65	.522	-0.23 (-1.17 0.31)	0.17

Results are expressed as mean ± SD (standard deviation). * $p_{Bonferroni\text{-}corrected} < 0.008$

Supplementary Figures

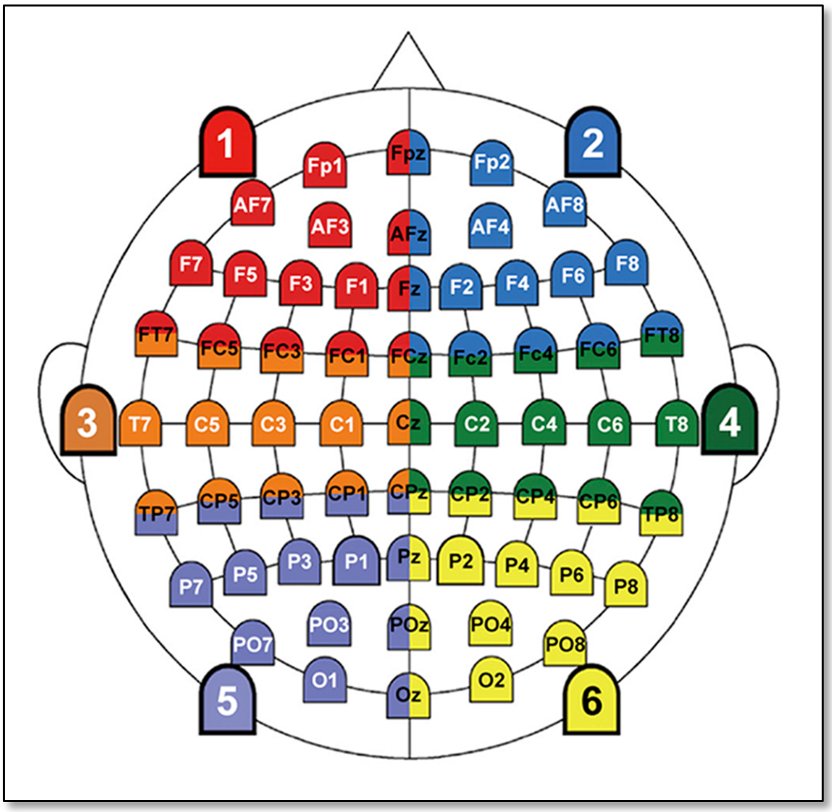

Figure S1. EEG montage. Scalp EEG electrodes were grouped into six regions. To obtain more stable spatial patterns, the electrodes in the border of two regions were included in both regions. Reprinted with permission from publication (Lu et al., 2015).

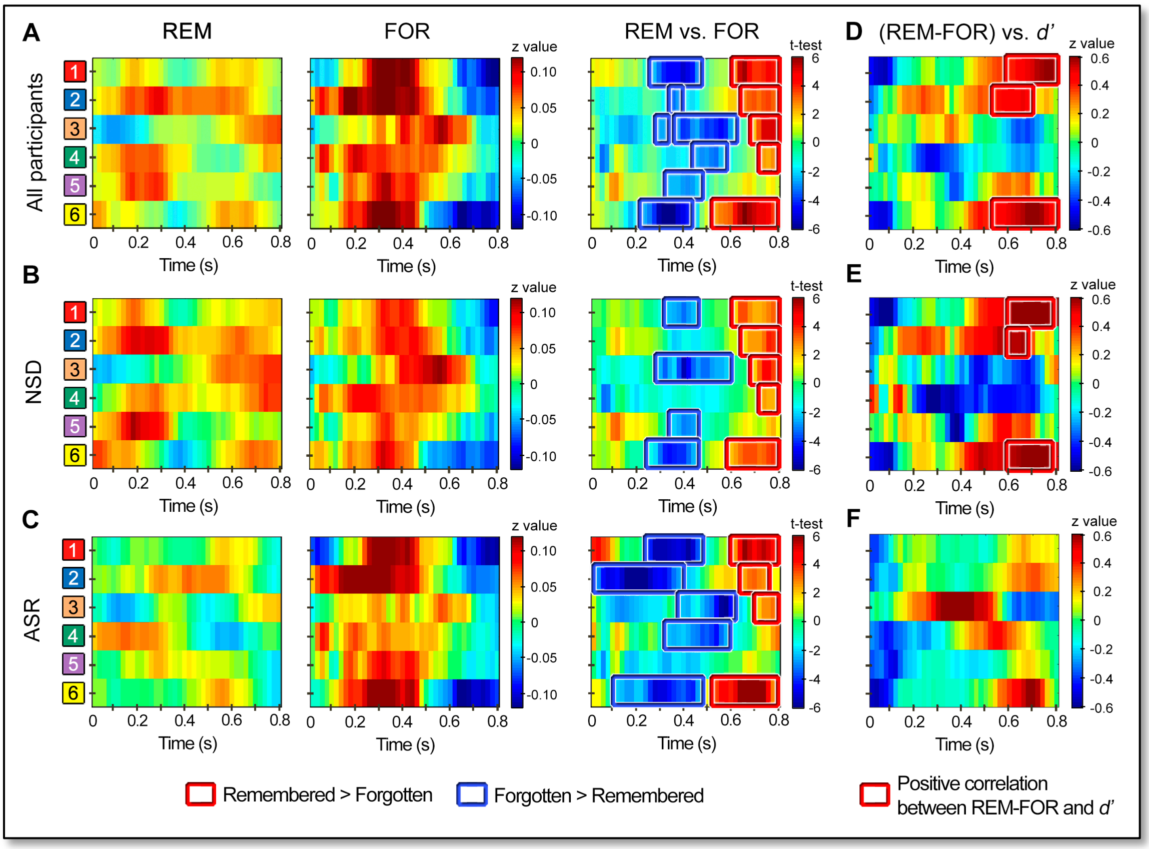

Figure S2. Contribution of encoding STPS after the 4th repetition of the event to memory recognition in the NSD and ASR group. Within-subjects STPS, expressed as averaged z-values, between the EEG activity elicited by the 3rd and 4th repetition of paired associates at encoding for subsequently remembered (REM) and forgotten (FOR) paired-associates in the recognition task across all participants (A) for the NSD group (B) and for the ASR group (C). The x-axis represents time, and the y-axis the spatial locations shown in Fig. 2. The statistics of contrasting STPS between remembered and forgotten paired-associates is shown in the third column. The red (REM>FOR) and blue squares (FOR>REM) refer to significant clusters resulting from comparing the two memory conditions. (D-F) The Pearson correlation coefficients converted to z scores between the STPS associated with the difference due to memory (REM-FOR) and the d' index across all participants (D), and across individuals of the NSD group (E) and ASR group (F). The red squares (positive correlation) refer to significant clusters resulting from such a correlation.

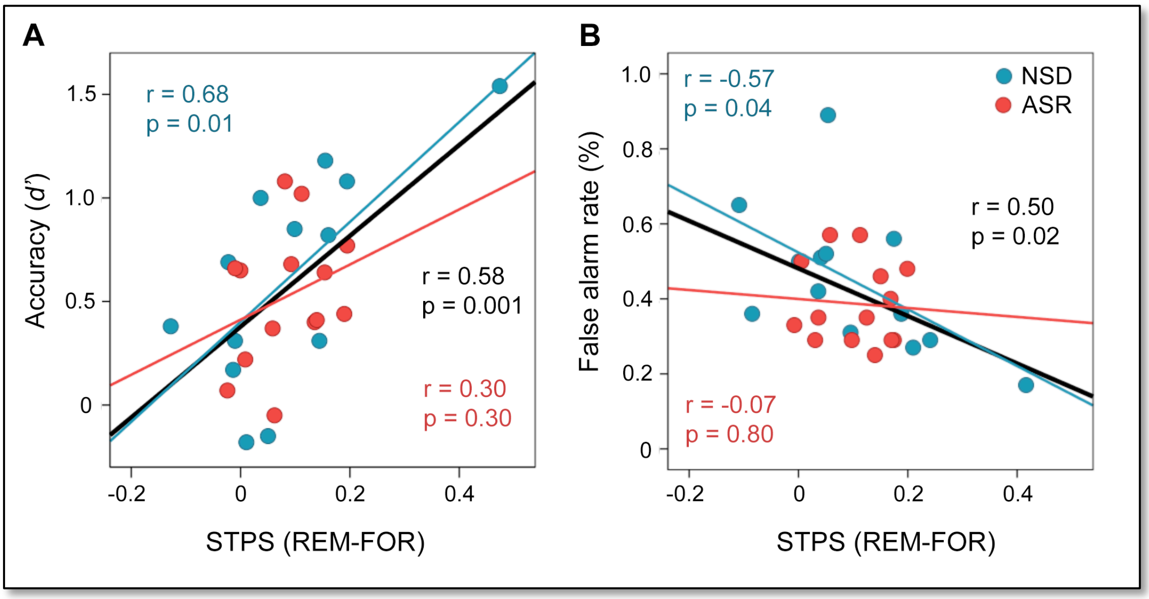

Figure S3. Relationship between the encoding STPS associated with the difference due to memory and recognition indices. (A) Scatter plot showing the correlation between the STPS (3rd vs. 4th repetition) associated with the difference due to memory and accuracy (*d'*) in the recognition task across all participants (black line) and for each group separately. (F) Scatter plot showing the correlation between the STPS (3rd vs. 4th repetition) associated with the difference due to memory and the false alarm rate in the recognition task across all participants (black line) and for each group separately.

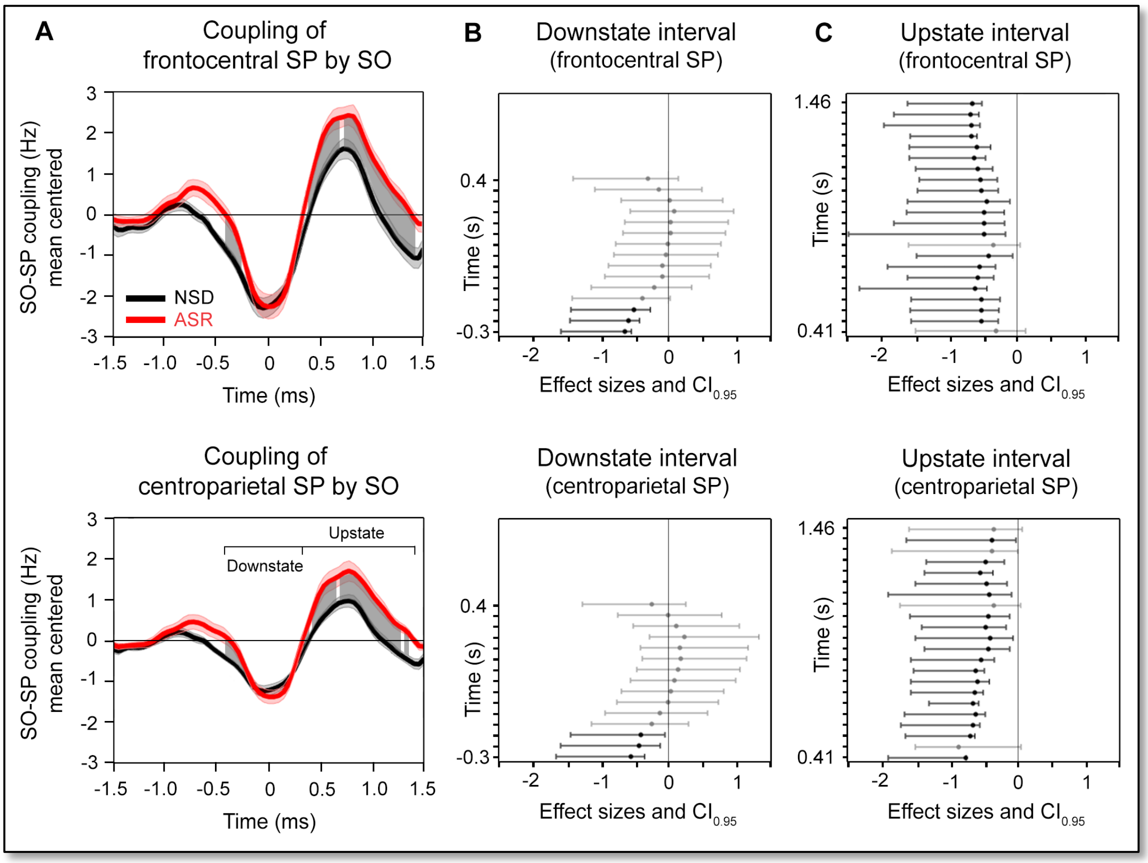

Figure S4. Effect of ASR on SO-SP coupling during SWS during the recovery sleep night. (A) Event correlation histogram between frontocentral SOs and fast SPs localized in frontocentral (top) and centroparietal electrodes (bottom) for the NSD group (black line) and ASR group (red line). Decreases in the number of SPs occurred in the SO down-state interval while increases occurred in the up-state interval. The vertical gray bars indicate between-group differences across time based on significant effect sizes. (B) Effect sizes (Hedges's *d*) and CI0.95 during the SO down-state for frontocentral (top) and centroparietal SPs (bottom). (C) The same as in B for the SO up-state. Note that most effect sizes were significant only in the up-state interval.

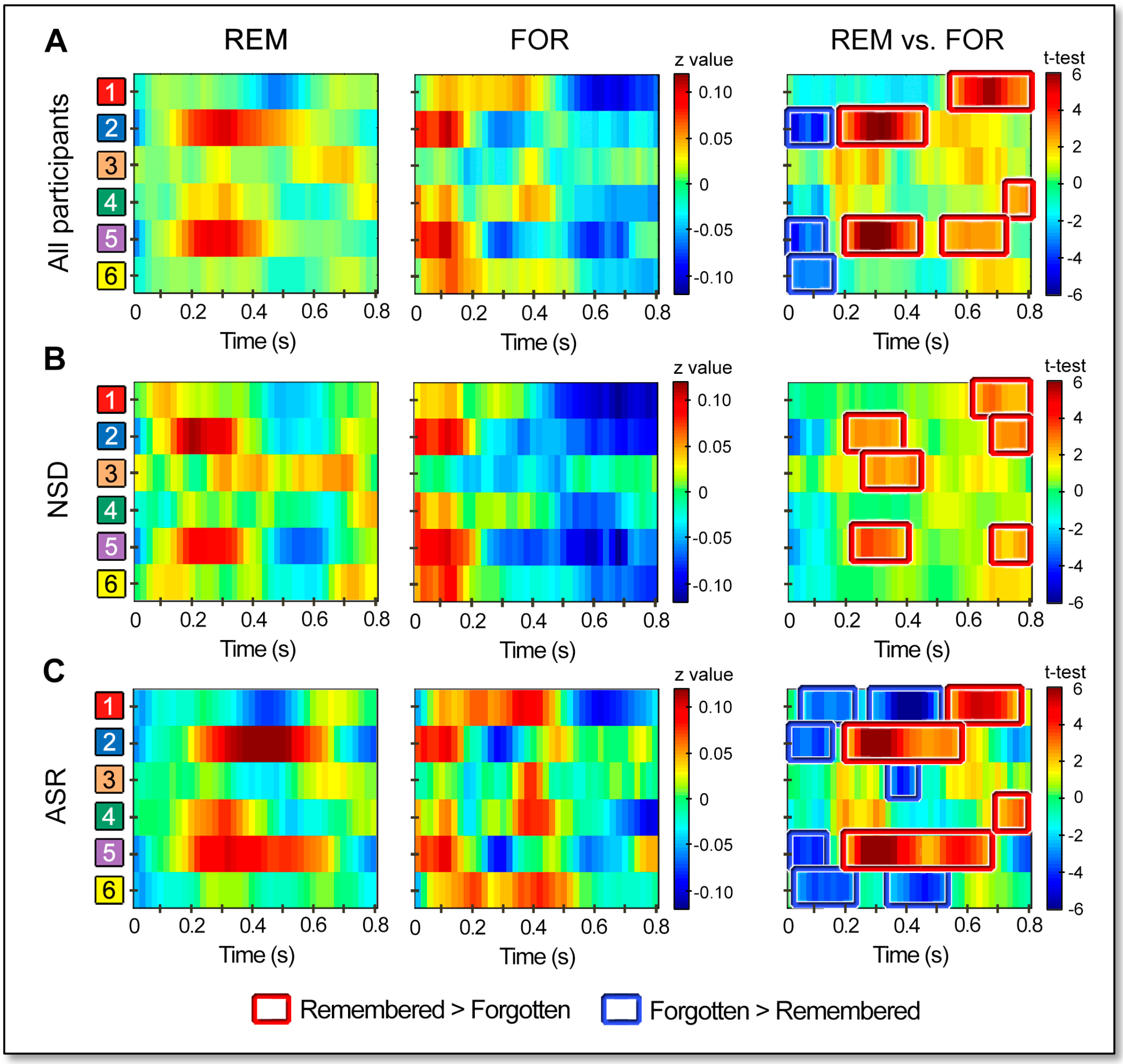

Figure S5. Contribution of encoding-retrieval STPS to subsequent memory. Within-subjects STPS, expressed as averaged z-values, between the EEG activity patterns elicited by the and 4th repetition of paired associates at encoding and the same pairs presented at retrieval for subsequently remembered (REM) and forgotten (FOR) paired-associates in the recognition task across all participants (A) for the NSD group (B) and for the ASR group (C). The x-axis represents time, and the y-axis the spatial locations shown in Fig. 2A. The statistics of contrasting STPS between remembered and forgotten paired-associates is shown in the right panel. The red and blue squares refer to significant clusters resulting from comparing the two memory conditions. The NSD group showed greater $STPS_{E\text{-}R}$ for remembered than for forgotten associations in two temporal clusters, one between 200-450 ms and the other between 650-800 ms, but only the former survived FWE correction. The cluster in the early time window was evident over right frontal and left centroparietal regions ($t_{(25)}$ = 3,72, $p_{cluster\text{-}corrected}$ = 0.039, d_{rm} $[CI_{0.95}]$ = 1.15 [0.61 1.85], *CL* = 0.85), and the cluster in the late time window emerged over frontal and left parietal regions ($t_{(25)}$ = 3.51, $p_{uncorrected}$ = 0.004, d_{rm} $[CI_{0.95}]$ = 1.26 [0.42 1.95], *CL* = 0.92). The ASR group also showed stronger $STPS_{E\text{-}R}$ for remembered compared with forgotten paired-associates in right frontal and left posterior areas, but for a longer period of

time ranging from 200 to 700 ms ($t_{(25)}$ = 9.78, $p_{cluster\text{-}corrected}$ = 0.0002, d_{rm} $[CI_{0.95}]$ = 3.40 [2.19 5.04], *CL* = 2.46). Contrary to the NSD group, reinstatement of frontal and right parietooccipital EEG patterns within the first 500 ms also contributed to recognition failure ($t_{(12)}$ = -6.96, $p_{cluster\text{-}corrected}$ = 0.017, d_{rm} $[CI_{0.95}]$ = 1.46 [0.77 2.21], *CL* = 1.16).

References

Andrillon, T. *et al*. Sleep spindles in humans: insights from intracranial EEG and unit recordings. *J. Neurosci.* **31**, 17821-17834 (2011).

Belenky, G. *et al.* Patterns of performance degradation and restoration during sleep restriction and subsequent recovery: a sleep dose-response study. *J. Sleep Res.* **12**, 1-12 (2003).

Cairney, S.A., Guttesen, A.Á.V., El Marj, N. & Staresina, B.P. Memory consolidation is linked to spindle-mediated information processing during sleep. *Curr. Biol.* **28**, 948-954 (2018).

Cohen, J. Statistical Power Analysis. *Curr. Dir. Psychol. Sci.* **1**, 98-101 (1992).

Cox, R., Gruber, G., Anderer, P., Asenbaum, S., Schimicek, P. & Saletu B. Large-scale structure and individual fingerprints of locally coupled sleep oscillations. *Sleep* **41**, zsy175 (2018).

Cox, R., Schapiro, A.C., Manoach, D.S. & Stickgold, R. Individual Differences in Frequency and Topography of Slow and Fast Sleep Spindles. *Front. Hum. Neurosci.* **11**, 433 (2017).

Craik, F.I.M. & Lockhart, R.S. Levels of processing framework for memory research. *J. Verb. Learn Verb. Behav.* **11**, 671-684 (1972).

Cumming, G. Understanding the New Statistics: Effect sizes, Confidence Intervals, and Meta-Analysis. New York, NY. Routledge (2012).

Fogel, S.M. & Smith, C.T. The function of the sleep spindle: a physiological index of intelligence and a mechanism for sleep-dependent memory consolidation. *Neurosci. Biobehav. Rev.* **35**, 1154-1165 (2011).

Heib, D.P. *et al.* Slow oscillation amplitudes and up-state lengths relate to memory improvement. *PLoS One*. 8, e82049 (2013).

Keppel, G. Design and Analysis: A researcher's handbook. Englewood Cliffs, NJ: Prentice Hall (1991).

Lakens, D. Calculating and reporting effect sizes to facilitate cumulative science: a practical primer for t-tests and ANOVAs. *Front. Psychol.* **4**, 863 (2013).

Leproult, R., Colecchia, E.F., Berardi, A.M., Stickgold, R., Kosslyn, S.M. & Van Cauter, E. Individual differences in subjective and objective alertness during sleep deprivation are stable and unrelated. *Am. J. Physiol. Regul. Integr. Comp. Physiol.* **284**, R280-R290 (2003).

Lu, Y., Wang, C., Chen, C. & Xue, G. Spatiotemporal neural pattern similarity supports episodic memory. *Curr. Biol.* **25**, 780-785 (2015).

Mander, B.A. *et al*. White Matter Structure in Older Adults Moderates the Benefit of Sleep Spindles on Motor Memory Consolidation. *J. Neurosci.* **37**, 11675-11687 (2017).

McGraw, K O. & Wong, SP. A common language effect size statistic. *Psychol. Bull.* **111**, 361-365 (1992).

Mei, N., Grossberg, M.D., Ng, K., Navarro, K.T. & Ellmore, T.M. Identifying sleep spindles with multichannel EEG and classification optimization. *Comput. Biol. Med.* **89**, 441-453 (2017).

Mölle, M., Marshall, L., Gais, S. & Born, J. Grouping of spindle activity during slow oscillations in human non-rapid eye movement sleep. *J. Neurosci.* **22**, 10941-10947 (2002).

Mölle, M., Bergmann, T.O., Marshall, L. & Born, J. Fast and slow spindles during the sleep slow oscillation: disparate coalescence and engagement in memory processing. *Sleep* **34**, 1411-1421 (2011).

O'Reilly, C., Warby, S.C. & Nielsen, T. Sleep Spindles: Breaking the Methodological Wall. Lausanne: Frontiers Media (2017).

Purcell, S.M. *et al*. Characterizing sleep spindles in 11,630 individuals from the National Sleep Research Resource. *Nat. Commun.* **8**, 15930 (2017).

Ray, L.B. *et al*. Expert and crowd-sourced validation of an individualized sleep spindle detection method employing complex demodulation an individualized normalization. *Front. Hum. Neurosci.* **9**, 507 (2015).

Staresina, B.P. *et al*. Hierarchical nesting of slow oscillations, spindles and ripples in the human hippocampus during sleep. *Nat. Neurosci.* **18**,1679-1686 (2015).

Urakami, Y., Ioannides, A.A. & Kostopoulos, G.K. Sleep spindles – as a biomarker of brain function and plasticity. In I. H. Ajeena (Ed.) Advances in clinical neurophysiology (chapter 4). (2012).

Werth, E., Achermann, P., Dijk, D.J. & Borbély, A.A. Spindle frequency activity in the sleep EEG: individual differences and topographic distribution. *Electroencephalogr. Clin. Neurophysiol.* **103**, 535-542 (1997).

Wu, H. *et al.* Effects of different sleep restriction protocols on sleep architecture and daytime vigilance in healthy men. *Physiol. Res.* 59, 821-829 (2010).

Zeitlhofer, J., Gruber, G., Anderer, P., Asenbaum, S., Schimicek, P., Saletu, B. Topographic distribution of sleep spindles in young healthy subjects. *J. Sleep Res.* **6**, 149-155 (1997).

Zhang, H., Fell, J. & Axmacher, N. Electrophysiological mechanisms of human memory consolidation. *Nat. Commun.* **9**, 4103 (2018).
